# Free-Living Aquatic Turtles as Sentinels of *Salmonella* spp. for Water Bodies

**DOI:** 10.3389/fvets.2021.674973

**Published:** 2021-07-22

**Authors:** Sonia M. Hernandez, John J. Maurer, Michael J. Yabsley, Valerie E. Peters, Andrea Presotto, Maureen H. Murray, Shannon Curry, Susan Sanchez, Peter Gerner-Smidt, Kelley Hise, Joyce Huang, Kasey Johnson, Tiffany Kwan, Erin K. Lipp

**Affiliations:** ^1^Warnell School of Forestry and Natural Resources, University of Georgia, Athens, GA, United States; ^2^Department of Population Health, Southeastern Cooperative Wildlife Disease Study, College of Veterinary Medicine, University of Georgia, Athens, GA, United States; ^3^Department of Population Health, Poultry Diagnostic and Research Center, College of Veterinary Medicine, University of Georgia, Athens, GA, United States; ^4^Department of Biological Sciences, Eastern Kentucky University, Richmond, KY, United States; ^5^Department of Geography, University of Georgia, Athens, GA, United States; ^6^Davee Center for Epidemiology and Endocrinology and the Urban Wildlife Institute, Lincoln Park Zoo, Chicago, IL, United States; ^7^Athens Veterinary Diagnostic Laboratory, College of Veterinary Medicine, University of Georgia, Athens, GA, United States; ^8^Enteric Diseases Laboratory Branch, Centers for Disease Control and Prevention, Atlanta, GA, United States; ^9^Department of Environmental Health Science, College of Public Health, University of Georgia, Athens, GA, United States

**Keywords:** chelonia, turtle, *Salmonella*, *Salmonella enterica*, reptile-associated salmonellosis

## Abstract

Reptile-associated human salmonellosis cases have increased recently in the United States. It is not uncommon to find healthy chelonians shedding *Salmonella enterica*. The rate and frequency of bacterial shedding are not fully understood, and most studies have focused on captive vs. free-living chelonians and often in relation to an outbreak. Their ecology and significance as sentinels are important to understanding *Salmonella* transmission. In 2012–2013, *Salmonella* prevalence was determined for free-living aquatic turtles in man-made ponds in Clarke and Oconee Counties, in northern Georgia (USA) and the correlation between species, basking ecology, demographics (age/sex), season, or landcover with prevalence was assessed. The genetic relatedness between turtle and archived, human isolates, as well as, other archived animal and water isolates reported from this study area was examined. *Salmonella* was isolated from 45 of 194 turtles (23.2%, range 14–100%) across six species. Prevalence was higher in juveniles (36%) than adults (20%), higher in females (33%) than males (18%), and higher in bottom-dwelling species (31%; common and loggerhead musk turtles, common snapping turtles) than basking species (15%; sliders, painted turtles). *Salmonella* prevalence decreased as forest cover, canopy cover, and distance from roads increased. Prevalence was also higher in low-density, residential areas that have 20–49% impervious surface. A total of 9 different serovars of two subspecies were isolated including 3 *S. enterica* subsp. *arizonae* and 44 *S. enterica* subsp. *enterica* (two turtles had two serotypes isolated from each). Among the *S. enterica* serovars, Montevideo (*n* = 13) and Rubislaw (*n* = 11) were predominant. *Salmonella* serovars Muenchen, Newport, Mississippi, Inverness, Brazil, and Paratyphi B. var L(+) tartrate positive (Java) were also isolated. Importantly, 85% of the turtle isolates matched pulsed-field gel electrophoresis patterns of human isolates, including those reported from Georgia. Collectively, these results suggest that turtles accumulate *Salmonella* present in water bodies, and they may be effective sentinels of environmental contamination. Ultimately, the *Salmonella* prevalence rates in wild aquatic turtles, especially those strains shared with humans, highlight a significant public health concern.

## Introduction

*Salmonella enterica* infections are a significant public health threat, responsible for over 93 million annual illnesses worldwide (1). In the United States alone, over 1 million cases of salmonellosis and 600 deaths are reported annually (2). Most cases of human salmonellosis are caused by food-borne *Salmonella* strains associated with contaminated meat, eggs, or produce. Produce has become a significant source of foodborne outbreaks associated with *Salmonella* (3–5), accounting for half the outbreaks and one quarter of the illnesses reported for the U.S. in 2016 alone (4). Water is central to growth and processing of fruits, vegetables, and nuts; and it is the most likely source of product contamination with *Salmonella* (6).

There has also been a significant number of human cases of salmonellosis linked to animal (4, 7–13) and environmental exposure (14–16) and a geographic disparity in reported cases of salmonellosis in the United States (17–19). Georgia has the highest annual *Salmonella* prevalence among the states; and within the state, the southern Coastal Plain has the highest incidence (20). There is a link between human cases and the Little River watershed in South Georgia, where 46% of *Salmonella* isolated from the Little River matched human isolates by pulsed-field gel electrophoresis (PFGE) (21). Understanding disease transmission in this region is difficult due to *Salmonella* strain diversity, its low abundance in water, and seasonal and weather-related fluctuations in its prevalence (14, 20, 21). Wildlife captured in the Little River watershed harbor some of the same *Salmonella* strains present in the river. However, only half of the raccoons and opossums sampled in this region possess the same *Salmonella* strains present in the CDC PulseNet database of human isolates (21). There are significant logistical challenges associated with sampling wildlife populations. Might an aquatic species prove a better sentinel for monitoring pathogenic *Salmonella* strains in watersheds or irrigation ponds?

For example, the American White Ibis (*Eudocimus albus*), an abundant aquatic bird, forms large nesting colonies in natural wetlands but have become habituated to living in agricultural and urban areas. Seventeen percent of ibis sampled in South Florida harbor *Salmonella* and 44% of these isolates match human isolates in the CDC-PulseNet database. Most notable was the spatial and temporal overlap in the isolation of these pathogenic strains with human cases of salmonellosis in South Florida. While this avian species is less likely to interact with humans and directly transmit *Salmonella* to people (11), it is a likely sentinel of environmental contamination (22). As a sentinel, the White Ibis is limited by its geographic distribution. Aquatic turtles, on the other hand, have a wide distribution across many different landscapes and habitat types, and often thrive in anthropogenic settings. Among wildlife, they are easy to capture, handle and sample.

Reptile-associated salmonellosis was a serious health problem in the 1960 and 1970s but was ameliorated, particularly in children, with public education and the 1975 federal ban of the sale of turtles <4 inches in carapacial length. In recent years, reptile-associated salmonellosis has increased again to comprise ~6% (74,000) of salmonellosis cases in the United States per year (24, 25). Most of these patients report contact with pet turtles (24, 26) and turtles have been responsible for several outbreaks of salmonellosis in the United States (8, 27, 28). Such outbreaks are typically associated with small turtles sold by street vendors and pet stores, despite the ban on their trade (29). Reptile-associated salmonellosis is most common in children (30, 31), and more likely to require hospitalization than other types of salmonellosis in other age groups (32). Understanding of turtle-associated salmonellosis primarily stems from epidemiological studies following outbreaks with reptile-associated serotypes (28) and surveys of captive turtles (33, 34). Several serotypes have been reported from turtles including the *S. enterica* serovars Muenchen, Typhimurium, Newport, Pomona, Litchfield and Paratyphi B. var L(+) tartrate positive (formerly Java) (8, 24–30). However, unique serovars are still being reported, e.g., *Salmonella* Agbeni (35), and there are many epidemiological gaps in understanding *Salmonella* carriage in turtles.

*Salmonella enterica* is routinely isolated from healthy, asymptomatic wild and pet chelonians (turtles and tortoises) and it is generally considered a normal component of their microbiota. The rate and frequency at which turtles shed *Salmonella*, and the conditions that may promote shedding, are not fully understood (36, 37). For commercial or pet turtles, hygiene, crowding, stress and other environmental factors may play a role in *Salmonella* shedding (38). This may, in part, explain the variability in past prevalence studies and may facilitate the role of turtles as *Salmonella* reservoirs for humans. Although some studies report low prevalence of *Salmonella* in free-ranging turtles, there is evidence that the prevalence can be higher in free-living turtles relative to captive turtles (39–42), likely influenced by species natural history (e.g., foraging behavior and habitat use), habitat quality, and other factors (e.g., landscape) that are largely unexplored. Several studies have investigated carriage of *Salmonella enterica* in free-living chelonians, with reported prevalence rates varying considerably depending on species, location and sampling methodology (40–45). To date, only a few studies have investigated the link between *Salmonella* prevalence in free-living turtles and human illness (7, 39, 46–48).

A better understanding of the public health risks of environmental exposure and the role of free-living turtles in transmission is especially important in regions where human salmonellosis is particularly high, such as the southeastern United States (19, 49). Of particular relevance, this region holds 10% of the world's aquatic turtle biodiversity (50). Aquatic turtles are ubiquitous throughout urban, suburban, and natural environments. In urban environments, they readily colonize ponds contaminated with runoff, often at high densities. Therefore, turtles are hypothesized to be good indicators of environmental contamination with *Salmonella*.

In 2012–2013, aquatic turtles from man-made ponds in north-central Georgia (Clarke and Oconee Counties) were surveyed for *Salmonella*. *Salmonella* prevalence by turtle species, basking ecology, demographics (age/sex), season, and landscape variables were investigated. Given the paucity of information regarding how various factors influence *Salmonella* prevalence in wild turtle populations, landscape variables related to water quality were examined to identify any associations with prevalence. Additionally, to better understand the role of wild turtles in non-foodborne human salmonellosis cases in Georgia, PFGE patterns were compared among *Salmonella* isolates recovered from wild turtles, archived animal, water, and human isolates.

## Materials and Methods

### Study Sites, Geographic Description, Animal Capture, and Sampling

**Figure 1:**
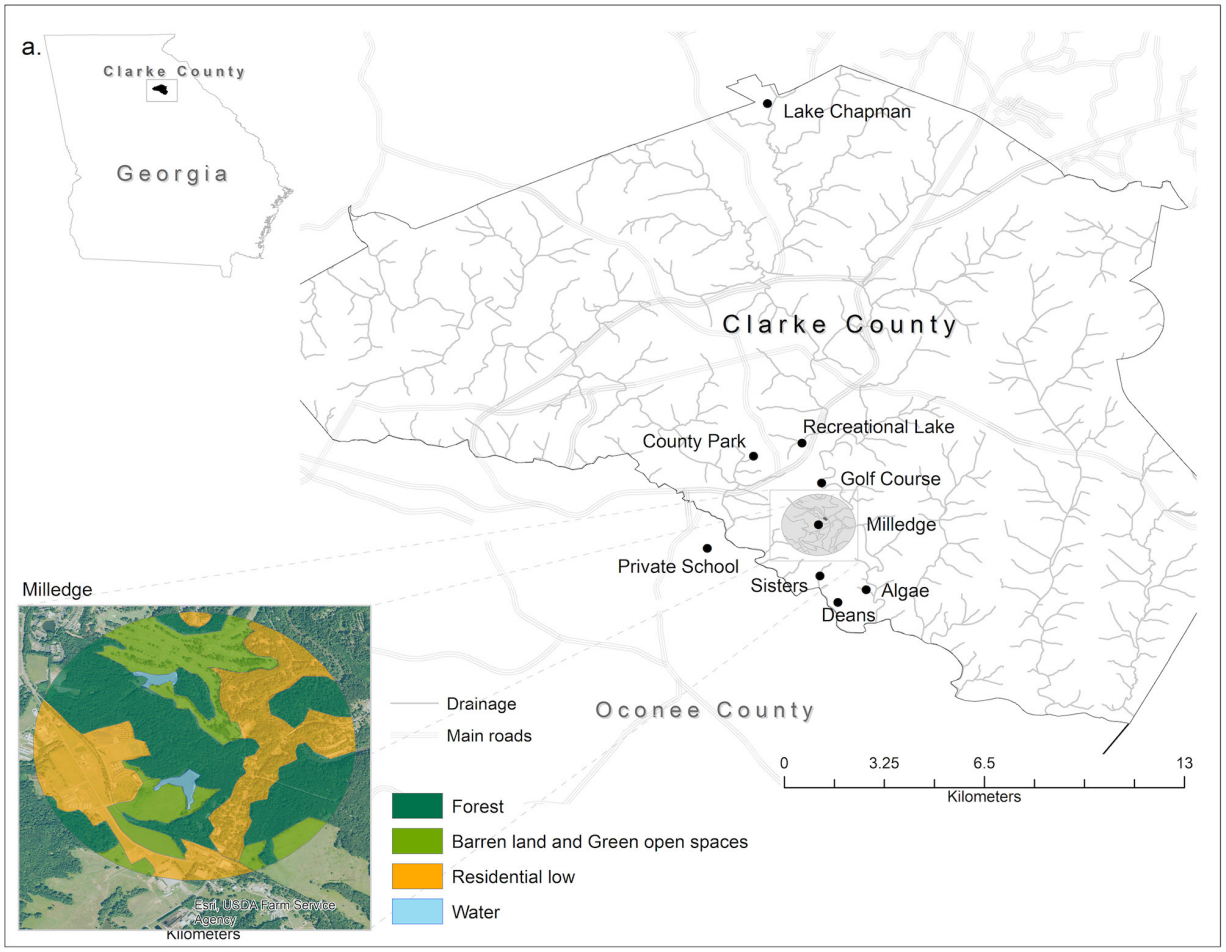
Map of the eight capture sites where turtles were captured for *Salmonella* testing. Seven sites were in Athens-Clarke County, Georgia (Algae Pond, Sisters Pond, Lake Chapman, UGA Golf Course, Milledge Pond, County Park, and Recreational Lake) and one was in Oconee County, Georgia (Private School). Inset shows and example of the land use categories surrounding each sample site. Land cover data are derived from the National Agriculture Imagery Program (NAIP, 2016) by the USDA's Farm Service Agency (FSA). Classes' denomination is based on the National Land Cover Classification. ArcGIS 10.5 licensed to Salisbury University, MD was used to extract the land cover features. All vector data used can be found freely available at TIGER from the U.S. Census Bureau Database.

Turtles were captured from April 2012 to June 2013 at eight small man-made ponds. Seven ponds were in Clarke County, Georgia (Algae Pond, Sisters Pond, Lake Chapman, UGA Golf Course, Milledge Pond, County Park, and Recreational Lake). Two of these ponds, Algae Pond and Sisters Pond, were located within the Whitehall Experimental Forest; the others were public or private ponds located on separate properties (Figure 1). The remaining pond was in Oconee County, Georgia at a private school. All water bodies were within the Oconee River watershed.

Turtles were captured using standard hoop traps (Memphis Net & Twine, Inc., Memphis, TN), baited with oil-packed sardines or herring and placed such that turtles could surface to breathe. Within each pond, traps were positioned in locations that were predicted to be the most successful: e.g., in areas that were shaded or more densely vegetated, or near substrates suitable for turtle basking such as exposed logs or rocks. Traps were placed in these locations for 2–4 days at a time, checked daily, and rebaited after 2 or 3 days. Once in hand, turtles were identified to species, measured (utilizing standard morphometrics for chelonians), and weighed. The age and sex of each individual was determined by species-specific morphological characteristics as described by Buhlmann et al. (51). Turtles were individually held in clean plastic containers until they defecated or overnight (maximum time needed for all to defecate). Feces were collected with sterile plastic pipettes and ~1 g of feces was suspended in 10 ml of dulcitol selenite (Difco; Detroit, MI). Turtles were subsequently released at the capture site. All containers were cleaned with soap and water and disinfected with a 10% bleach solution before they were reused. All fecal samples in selenite were submitted on the same day of collection to the Athens Diagnostic Laboratory (Athens, GA) for culture. All animal capture and animal handling procedures were approved by the University of Georgia's Institutional Animal Care and Use Committee (AUP# A2010 10-186).

### *Salmonella* Isolation and PFGE Molecular Characterization

Feces in selenite was incubated overnight at 42°C for *Salmonella* enrichment (52). A 10 μl loopful of the overnight enrichment was plated onto xylose lysine deoxycholate (XLD) and brilliant green (BG) plates (Remel Inc., Lenexa, KS) and incubated overnight at 37°C as previously described (10, 53). H_2_S-positive, black colonies were picked and subcultured onto blood agar plates (tryptic soy agar with 5% sheep blood). Final *Salmonella* confirmation was determined with the following tests on a single isolated colony: citrate, triple sugar iron (TSI), and motility-indole-ornithine media (Beckton and Dickson, Franklin Lakes, NJ); and a whole-cell agglutination test using *Salmonella*-specific poly A-I and Vi antiserum (Fisher Scientific, Pittsburgh, PA). Microbial identification as *Salmonella* was based on possessing all of the following criteria. *Salmonella* grows on TSI slant producing a red slant, yellow/black (H_2_S-production) butt, and gas. In addition, *Salmonella* is motile, citrate-positive, indole and ornithine negative and agglutinates with poly A-I/Vi antiserum (54). Samples were considered culture negative if no black or pink colonies were observed on XLD or BG sections, respectively. A delayed-secondary enrichment was done for samples that were culture negative after the primary enrichment and initial plating on XLD and BG. A 10 μl loopful of the secondary enrichment in selenite overnight was plated onto XLD and BG plates. *Salmonella* identification of suspect colonies was confirmed as previously stated. Isolates were forwarded to the National Veterinary Service Laboratory (NVSL) at Ames, Iowa, for definitive *Salmonella* serotyping.

At the time of sample submission, PFGEs were still the primary method utilized by the CDC to determine genetic relatedness among *Salmonella* isolates by comparison with human isolates in the CDC PulseNet USA national database. Agarose plugs and PFGE conditions were performed as previously described (53, 55–57). Electrophoresis was done using the CHEF DR II electrophoresis unit (Bio-Rad; Hercules, CA), with 0.5X Tris-borate-EDTA buffer (Sigma-Aldrich; St. Louis, MO); 6 V/cm with pulse times 2.25–63.85 s at 14°C for 15.5 h. A master database of *Salmonella* PFGE patterns in BioNumerics (Applied Maths; Austin, TX) contains over 1,000 PFGE entries for *Salmonella* isolated from water and various animal species (10, 11, 21). Comparisons were made between PFGE patterns in BioNumerics using Dice coefficient and unweighted pair group method of arithmetic averages (UPGMA) clustering. Clusters were based on a 75% similarity cut-off (21). Turtle isolates were also compared to archived isolates previously acquired from animal and water samples from the Oconee River watershed (21).

### Landscape Data

All landscape data were public and freely available on government databases. The geographic coordinates of each pond were collected using a GPS hand device during the turtle captures. From each GPS location, a buffer of 1 km was established. Land cover data was extracted from the National Agriculture Imagery Program (NAIP-USDA) and was classified based on the National Land Cover Classification system (58), applied to the state level. The NAIP ortho-corrected imagery was used because the high spatial resolution of NAIP was suitable for a more precise land cover classification at 1 km around the ponds. NAIP imagery was classified based on the national land cover classification system (United States Geological Survey) (58). Within each 1 km buffer, the summarized land cover classes accounted for 100% of all classes surrounding that pond. For instance, the Low Intensity Urban areas were defined as 20–49% of impervious surfaces, which most commonly included single-family housing units (59). The land cover classifications utilized and the percentages of each class per pond are summarized on Supplementary Material. The National Road System data were used to measure the pond distances to the road types. Road types and human population were collected at the Topologically Integrated Geographic Encoding and Referencing database—TIGER, U.S. Census Bureau Database. ArcMap 10.6 (60) was used to extract the land cover, measure the distance from pond to features and measure the areas of each land cover class.

### Statistical Analyses

*Salmonella* prevalence rates in turtles were calculated as the number of individual turtles with a *Salmonella* shedding status of positive divided by the total number of turtles captured and tested (all turtles captured regardless of *Salmonella* status). *Salmonella* prevalence rates were analyzed using generalized linear mixed effects models in R version 3.3.1 (61) with the *lme4* package (62). The response variable, *Salmonella* prevalence, was modeled using a binomial error distribution, and all models included the pond from which turtles were sampled as a random effect. Likelihood ratio tests were used to test the significance of the following predictor variables for *Salmonella* prevalence rates: turtle species, turtle basking ecology (basking vs. non-basking species), pond area size, distance (meters) of pond to the closest highway or street (tested separately), and the percentages of (a) canopy cover over pond, (b) forest cover over pond, (c) low density residential land around each pond, (d) water, and green open space. All percentages were calculated at the 1 km landscape around the pond. The likelihood ratio test uses a chi-square distribution to determine the contribution of a single factor by comparing the fit of the model with and without the factor of interest (63). Chi-square analysis was used to examine differences in *Salmonella* prevalence between age and sex classes.

A candidate set of 11 single-factor and two-factor models were tested using an AICc model selection approach to understand which factors at the local and landscape scale influenced *Salmonella* prevalence rates in turtles (Table 1). The candidate set included a subset of the above listed predictor variables based on *a priori* hypotheses about the factors posited to influence *Salmonella* in turtles. The null model, including only the random effect of pond, was included in the candidate set.

**Table 1:** Selection parameters of candidate generalized linear mixed models explaining variation in *Salmonella* prevalence rates of turtles.

**Candidate models**	***df***	**AICc**	**ΔAICc**	**AICc (*wi*)**	***R*^**2**^**
**BaskingEcology**	**3**	**202.78**	**0.00**	**0.23**	**0.083**
Basking ecology + Forest cover	4	203.20	0.42	0.19	0.100
Basking ecology + Distance highway	4	203.21	0.42	0.19	0.095
Basking ecology + Canopy cover	4	204.07	1.29	0.12	0.089
Basking ecology + Low resid cover	4	204.68	1.90	0.09	0.084
Basking ecology + Distance street	4	204.73	1.95	0.09	0.084
Basking ecology + Area pond	4	204.87	2.09	0.08	
Distance highway	3	210.26	7.47	0.01	
Null	2	210.76	7.98	0.00	
Distance highway + Low resid cover	4	211.53	8.75	0.00	
Canopy cover quadratic	3	212.68	9.90	0.00	

*All models use a binomial error distribution and location as a random effect. For each model, df, degrees of freedom, AICc, AIC corrected from small sample size, change in AICc (ΔAICc), AICc weight AICc (wi), and deviance explained (R^2^) for the best and competing models are shown. The best fit model is shown in bold*.

## Results

**Figure 2:**
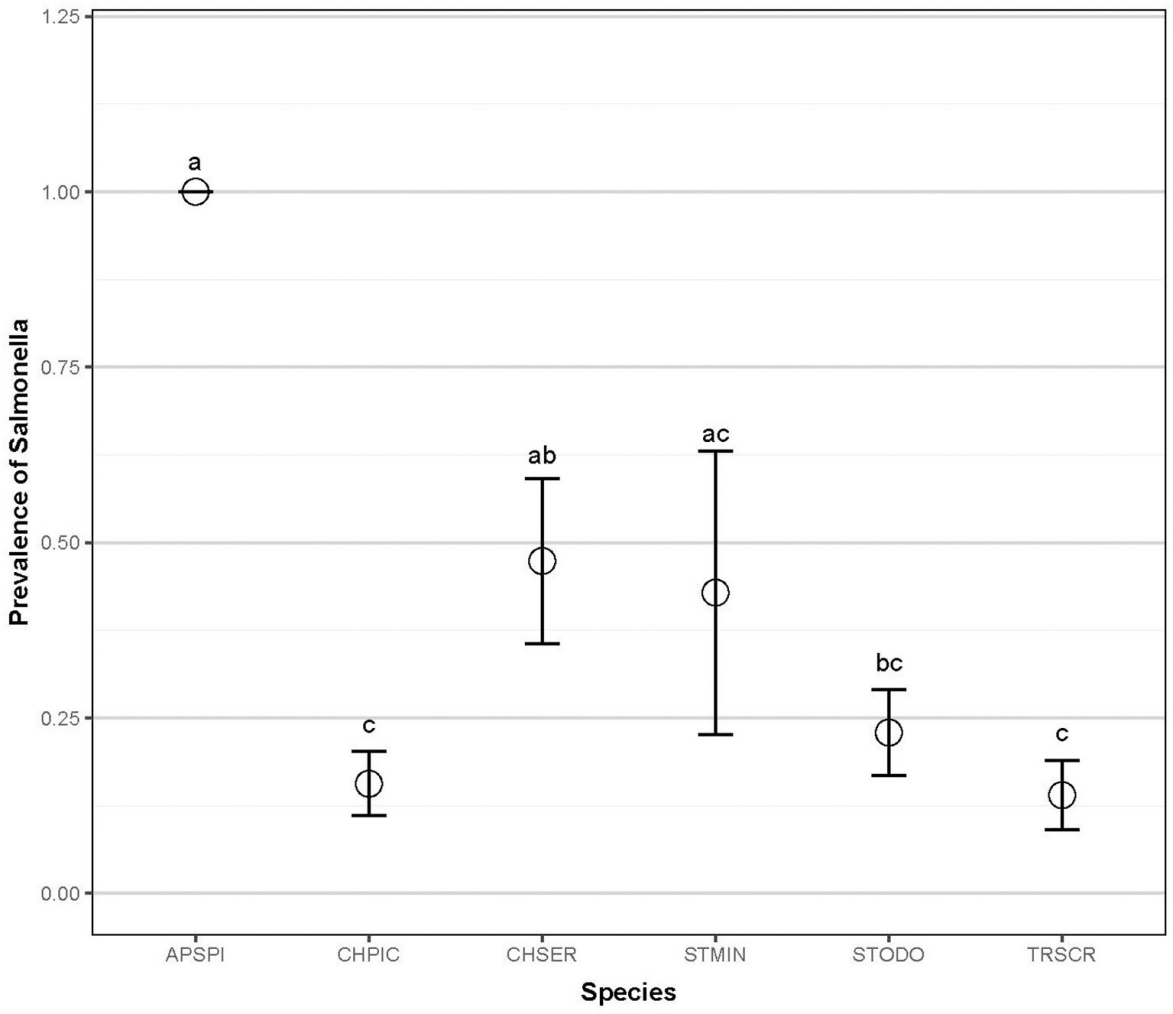
*Salmonella* prevalence of six aquatic turtle species (2012–2013). Letters indicate statistical significance. Common snapping turtle, *Chelydra serpentina* (CHSER, *n* = 20); common musk turtle, *Sternotherus odoratus* (STODO, *n* = 48)*;* Eastern painted turtle, *Chrysemys picta* (CHPIC, *n* = 65); yellow-bellied slider, *Trachemys scripta scripta* (TRSCR, *n* = 50); Spiny softshell turtle, *Apalone spinifera* (APSPI, *n* = 4); and loggerhead musk turtle, *Sternotherus minor* (STMIN, *n* = 7).

Fecal samples were collected from 194 individual wild turtles representing six species: common snapping turtle, *Chelydra serpentina* (CHSER, *n* = 20); common musk turtle, *Sternotherus odoratus* (STODO, *n* = 48); Eastern painted turtle, *Chrysemys picta* (CHPIC, *n* = 65); yellow-bellied slider, *Trachemys scripta scripta* (TRSCR, *n* = 50); spiny softshell turtle, *Apalone spinifera* (APSPI, *n* = 4); and loggerhead musk turtle, *Sternotherus minor* (STMIN, *n* = 7). In total, *Salmonella* was isolated from 45 of the sampled turtles (23.2%). *Salmonella* prevalence between species ranged from 14 to 100%: snapping (45 ± 0.25, 95%CI), common musk (22.9 ± 0.12%), painted (16.9 ± 0.09%), slider (14 ± 0.10%), softshell (100 ± 0.00%), and loggerhead musk (42.8 ± 0.50%; Figure 2; Chisq = 23.20; *p* < 0.001). *Salmonella* prevalence in softshell turtles was significantly higher than in painted, common musk, and sliders, and the prevalence in painted turtles was significantly lower than in softshell and snapping turtles (Figure 2).

**Figure 3:**
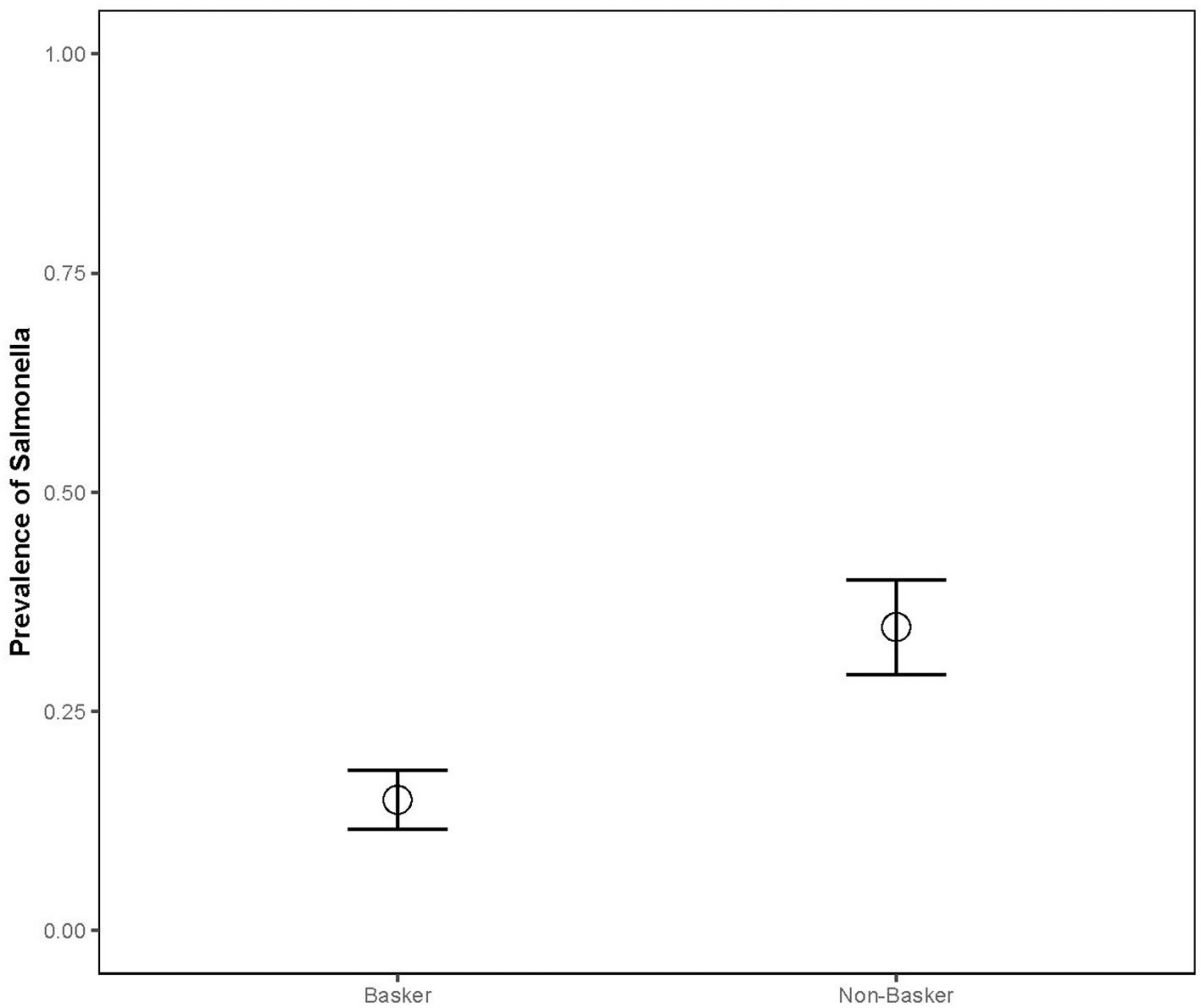
*Salmonella* prevalence of aquatic turtle species varied based on their basking behavior (2012–2013). CHPIC and TRSCR were classified as baskers, while CHSER, STODO, and STMIN were classified as non-baskers. APSPI were not included in basking analysis due to sample size and inconsistent basking behavior.

**Figure 4:**
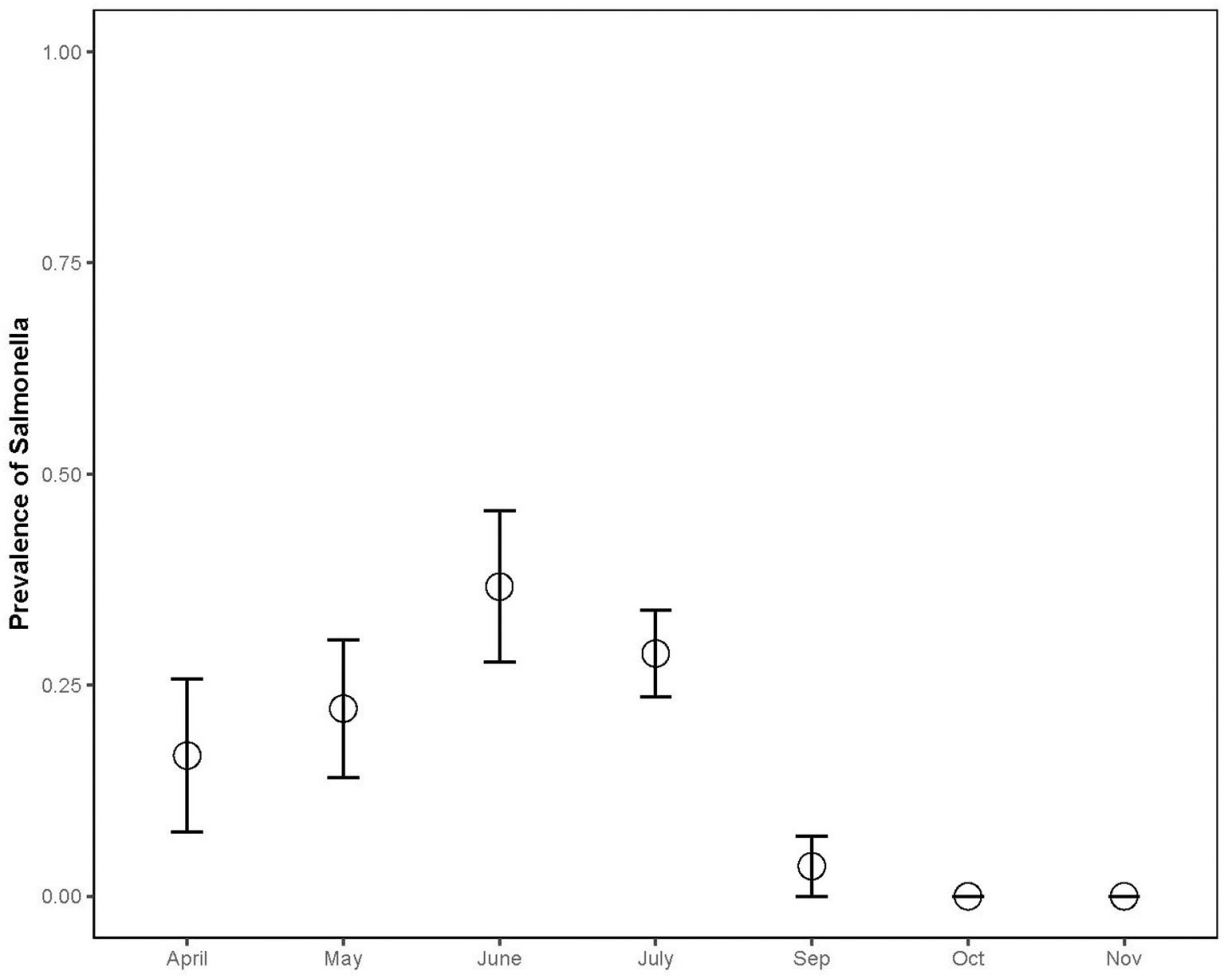
*Salmonella* prevalence of aquatic turtle species varied by sampling month (2012-2013).

The prevalence in juveniles (*n* = 25, 36%) was significantly higher than in adults (*n* = 124, 20%) (Chisq = 5.884, *p* = 0.0153), and the prevalence in bottom-dwelling species (*n* = 78, 31%) (common and loggerhead musk and snapping turtles) was significantly higher than in basking species (*n* = 114, 15%; Chisq = 10.04; *p* = 0.001; Figure 3) (sliders and painted turtles). The prevalence for females (*n* = 63, 33%) was significantly higher than males (*n* = 93, 18%) (Chisq = 4.006, *p* = 0.045). The combined prevalence rates of *Salmonella* during the capture months for both sampling years were as follows: April (*n* = 20, 17%), May (*n* = 27, 22%), June (*n* = 29, 37%), July (*n* = 79, 29%), September (*n* = 28, 4%), October (*n* = 7, 0%), and November (*n* = 2, 0%) (Chisq = 17.83; *p* = 0.007; Figure 4). *Post-hoc* pairwise comparisons did not find significant differences between months, but prevalence did increase with increasing ambient temperatures, inferred from seasonal monthly data.

The best model explaining *Salmonella* prevalence rates in turtles (23% support) included only the single predictor of “basking ecology” (Table 1). The candidate set of models contained additional supported models that included basking class, plus a land cover variable. Two of these competing models had 19% support; one model included the effect of basking class and percent forest cover in the landscape, and the other included the effect of basking class and the distance of the pond from the closest highway. In both of these models, landscape variables had a negative effect on the prevalence of *Salmonella* in turtle populations, in other words, as forest cover increased in the landscape, there was a lower prevalence of *Salmonella* in the turtles, and the closer a pond was to the highway, the higher the prevalence of *Salmonella*. A third competing model with 12% support included the effect of basking class and the percent canopy cover over the pond sampled. As canopy cover increased over the pond, the prevalence of *Salmonella* in the turtle population decreased. Finally, two competing models had 9% support; one model included the effect of basking class and percent cover of low-density residential land in the landscape, and the other model included the effect of basking class and the distance of the pond to the closest street. As the percent cover of low-density residential area in the landscape increased, the prevalence of *Salmonella* in turtle populations increased. In addition, the distance to the closest street had a negative effect on *Salmonella* prevalence (i.e., the closer the pond was to the street, the higher the *Salmonella* prevalence).

**Figure 5:**
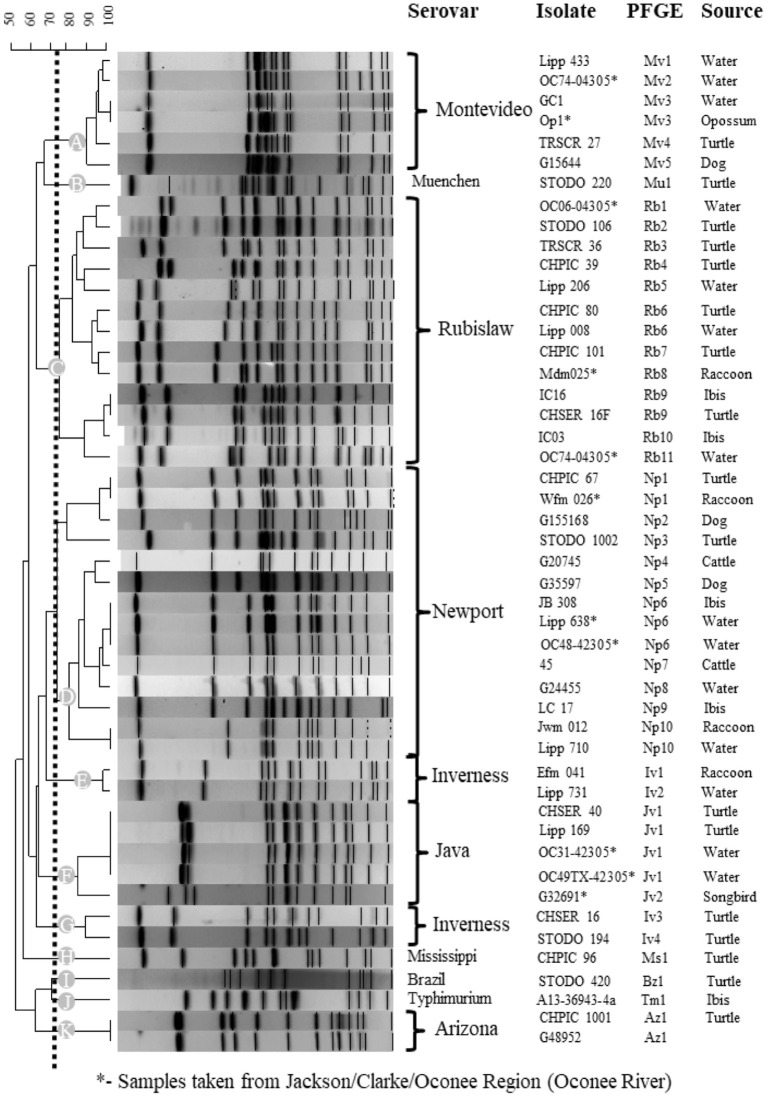
Genetic relatedness of *Salmonella enterica* isolated from turtles, other animal species and water collected from the Oconee River watershed and Athens-Clarke Georgia. Pulsed gel-electrophoresis (PFGE) was used to determine genetic relatedness among turtle isolates and archived animal and water isolates. A subset of similar or matching *Salmonella* PFGE patterns (>75%) are presented for the over 1,000 PFGE entries in the BioNumerics database. Level of similarity was calculated by the band-based Dice coefficient. Clustering of samples was performed using the unweighted pair-group method with arithmetic averaging (UPGMA) to generate this dendrogram. Dotted line illustrates the 75% cutoff used to identify similar and matching PFGE patterns and the 11 clusters (A-K) identified in this analysis. * indicates PFGE patterns for *Salmonella* isolates collected from the Oconee River watershed which runs through Athens-Clarke county, Georgia, and neighboring Jackson and Oconee counties.

A total of nine different serovars of two subspecies were isolated from turtles including 3 *S. enterica* subsp. *arizonae* and 44 *S. enterica* subsp. *enterica* (Table 2). Two turtles had two serotypes isolated from each individual, one with Rubislaw and Muenchen and another with Newport and Mississippi. Among the *S. enterica* serovars, two (Montevideo (*n* = 13) and Rubislaw (*n* = 11) were predominant, and fewer numbers of serovars Muenchen, Newport, Mississippi, Inverness, Brazil, and Paratyphi B. var L(+) tartrate positive (Java) were isolated (Table 2). *Salmonella enterica* IIIa Arizonae, a subspecies commonly isolated from reptiles (64, 65), was only isolated from three turtles (6.7% of the total turtles positive for *Salmonella*). Five of the nine *Salmonella* serovars isolated from turtles were previously isolated from wildlife and water in the Oconee River watershed (Table 2) (21). PFGE patterns of *Salmonella* isolates clustered into 16 types indicating genetic relatedness. These clusters were unique for each serovar, with the exception of *S*. Inverness which consisted of two distinct PFGE clusters, E and G (Figure 5). *Salmonella* Rubislaw had the greatest diversity of PFGE types (Table 2). Of the 16 PFGE types found in turtles, five were previously found in animal and water samples from the Oconee watershed. The diversity in PFGE patterns was lower in *S. enterica* isolated from turtles compared to the same *Salmonella* serovars in the aforementioned samples from the Oconee River watershed (21, 66): 16 vs. 50, respectively (Table 2). Two *Salmonella* strain types, Muenchen (Mu1) and Montevideo (Mv4), were the most common PFGE types identified in turtle isolates: 13.3 and 28.9%, respectively.

**Table 2:** *Salmonella enterica* serovar and strain diversity among turtles sampled in Athens, Georgia.

***Salmonella enterica* subspecies/Serovar**	**No**.	**PFGE type**	**No. of specific PFGE type**	**Total PFGE types (Turtles)**	**Total PFGE types (Oconee river watershed)^**a**^**
*S. enterica* IIIa Arizonae; 51:z4,z23:-	3	Az1	3	1	
*S. enterica* I; Brazil	2	Bz1	2	1	
*S. enterica* I; Inverness	4	Iv3	2	2	
		Iv4	2		
*S. enterica* I; Java^b^^,^ ^c^	1	Jv1^b^^,^ ^d^^,^ ^e^	1	1	2
*S. enterica* I; Mississippi	3	Ms1	3	1	
*S. enterica* I; Montevideo^b^^,^ ^c^	13	Mv4	13	1	4
*S. enterica* I; Muenchen^b^^,^ ^c^	7	Mu1	7	1	25
*S. enterica* I; Newport^b^^,^ ^c^	3	Np1^b^	2	2	5
		Np3	1		
*S. enterica* I; Rubislaw^b^^,^ ^c^	11	Rb2	2	6	14
		Rb3	2		
		Rb4	1		
		Rb6	3		
		Rb7	1		
		Rb9	1		
Total	47^f^		46^g^	16	50

a *Diversity of Salmonella strain types isolated from Oconee River watershed; years 2005–2011 (21).*

b *Serovar or strain type isolated from Oconee River watershed.*

c *Salmonella serovars isolated multiple times from Oconee River watershed.*

d *Salmonella strain type isolated from Oconee River watershed.*

e *Salmonella strain type isolated from multiple species and sites.*

f *Two turtles were positive for two serotypes each, one with Rubislaw and Muenchen and another with Newport and Mississippi.*

g *One isolate was not included in the PFGE analysis*.

The majority of turtle isolates (40/46; 86.9%) had PFGE patterns that matched human cases in the CDC PulseNet USA database (Table 3). Fifty-one percent of the turtle isolates that matched human PFGE patterns (*n* = 20) in the PulseNet database were outbreak-related strains. Two-thirds of the turtle isolates had PFGE patterns that matched temporally or spatially with human cases reported from Georgia (Table 3). *Salmonella* strain types Jv1 (Java) and Ms1 (Mississippi) were also reported among human cases in Athens, Georgia (2009, 2016–2018).

**Table 3:** Matching Pulsed Field Gel Electrophoresis (PFGE) Patterns between *Salmonella enterica* isolated from turtles and humans.

**Isolate**	**Source**	**Locale^**a**^**	**Subspecies; Serovar**	**PFGE Pattern**	**PulseNet Pattern**
CHSER 40	CST	Lake Chapman	I; Java	Jv1^b^^,^ ^c^	JKXX01.0059^d−f^
CHPIC 105A	PT	Lake Chapman	IIIa Arizonae; 51:z4,z23:-	Az1	JR3X01.0005
CHPIC 101	PT	Lake Chapman	I; Rubislaw	Rb7	No matches
STODO 60	CMT	County Park	I; Rubislaw	Rb6^c^	JLPX01.0030
APSPI 53	SST	Lake Herrick	I; Montevideo	Mv4	JIXX01.0080^d^^,^ ^e^
APSPI 55	SST	Lake Herrick	I; Montevideo	Mv4	JIXX01.0080^d^^,^ ^e^
APSPI 54	SST	Lake Herrick	I; Montevideo	Mv4	JIXX01.0080^d^^,^ ^e^
APSPI 56	SST	Lake Herrick	I; Montevideo	Mv4	JIXX01.0080^d^^,^ ^e^
STODO 1002	CMT	Milledge Pond	I; Newport	Np3	JJPX01.0872^e^
CHSER 100 mp	CST	Milledge Pond	IIIa Arizonae; 51:z4,z23:-	Az1	JR3X01.0005
CHSER 57	CST	Milledge Pond	I; Montevideo	Mv4	JIXX01.0080^d^^,^ ^e^
CHSER 58	CST	Milledge Pond	I; Montevideo	Mv4	JIXX01.0080^d^^,^ ^e^
CHSER 24	CST	Milledge Pond	I; Rubislaw	Rb3	No matches
CHPIC 96	PT	Milledge Pond	I; Mississippi	Ms1	JIPX01.0007^d−f^
CHPIC 47	PT	Milledge Pond	I; Montevideo	Mv4	JIXX01.0080^d^^,^ ^e^
CHPIC 48	PT	Milledge Pond	I; Montevideo	Mv4	JIXX01.0080^d^^,^ ^e^
CHPIC 67	PT	Milledge Pond	I; Newport	Np1^b^	JJPX01.0507^d^^,^ ^e^
CHPIC 76	PT	Milledge Pond	I; Newport	Np1^b^	JJPX01.0507^d^^,^ ^e^
CHPIC 80	PT	Milledge Pond	I; Rubislaw	Rb6^c^	JLPX01.0030
CHPIC 76	PT	Milledge Pond	I; Rubislaw	Rb6^c^	JLPX01.0030
TRSCR 49	ST	Milledge Pond	I; Montevideo	Mv4	JIXX01.0080^d^^,^ ^e^
TRSCR 50	ST	Milledge Pond	I; Montevideo	Mv4	JIXX01.0080^d^^,^ ^e^
TRSCR 36	ST	Milledge Pond	I; Rubislaw	Rb3	No matches
STODO14	CMT	Golf Course	I; Montevideo	Mv4	JIXX01.0080^d^^,^ ^e^
STODO	CMT	Golf Course	I; Inverness	Iv3	JRLX01.0031^e^
CHSER 16F	CST	Golf Course	I; Rubislaw	Rb9	JLPX01.0273
CHSER 16	CST	Golf Course	I; Inverness	Iv3	JRLX01.0031^e^
CHPIC 39	PT	Golf Course	I; Rubislaw	Rb4	JLPX01.0125
TRSCR 44	ST	Golf Course	I; Mississippi	Ms1	JIPX01.0007^d−f^
TRSCR 27	ST	Golf Course	I; Montevideo	Mv4	JIXX01.0080^d^^,^ ^e^
TRSCR 28	ST	Golf Course	I; Montevideo	Mv4	JIXX01.0080^d^^,^ ^e^
TRSCR 29	ST	Golf Course	I; Montevideo	Mv4	JIXX01.0080^d^^,^ ^e^
CHPIC 1001	PT	Algae Pond	IIIa Arizonae; 51:z4,z23:-	Az1	JR3X01.0005
STODO 106	CMT	Deans Pond	I; Rubislaw	Rb2	No matches
CHSER 100F	CST	Deans Pond	I; Rubislaw	Rb2	No matches
STODO 194	CMT	Lower Sisters Pond	I; Inverness	Iv4	No matches
STODO 2000	CMT	Lower Sisters Pond	I; Inverness	Iv4	No matches
STODO 01	CMT	Lower Sisters Pond	I; Java	Jv1^b^^,^ ^c^	JKXX01.0059^d−f^
STODO 420	CMT	Private School	I; Brazil	Bz1	Unnamed Pattern
STODO 220	CMT	Private School	I; Muenchen	Mu1	JJ6X01.0431^e^
STODO 450	CMT	Private School	I; Muenchen	Mu1	JJ6X01.0431^e^
CHSER 100	CST	Private School	I; Muenchen	Mu1	JJ6X01.0431^e^
CHSER 110	CST	Private School	I; Muenchen	Mu1	JJ6X01.0431^e^
STMIN 300	SMT	Private School	I; Brazil	Bz1	Unnamed Pattern
STMIN 110	SMT	Private School	I; Muenchen	Mu1	JJ6X01.0431
STMIN 30	SMT	Private School	I; Muenchen	Mu1	JJ6X01.0431

a *Sampling sites are ordered in this table geographically from north to south.*

b *Match with Salmonella strain isolated from the Oconee River.*

c *Litter River (21).*

d *Outbreak strain.*

e *Temporal overlap with human cases in Georgia reported for years 2012 or 2013.*

f *Human cases reported in Athens-Clarke county GA for the years 2009, 2016–2018. CSN, Common Snapping Turtle; PT, Painted Turtle; CMT, Common Musk Turtle; SST, Spiny Softshell turtle; ST, Slider Turtle; SMT, Stripeneck/Loggerhead Musk Turtle*.

## Discussion

Wild turtles in the United States are often presumed to harbor a high prevalence of *Salmonella* because most of the published information regarding *Salmonella* prevalence in turtles comes from studies focused primarily on pet turtles or from epidemiological investigations following an outbreak, which often involve commercial breeding facilities (28, 67–69). Compared to studies in other countries (7, 34, 39, 40, 43, 45, 47, 70–73), there is a paucity of information about *Salmonella* prevalence in wild turtles, in the United States (41, 42, 44, 74). This study attempts to fill that knowledge gap and demonstrated a wide range of prevalence of *Salmonella* among free-living turtle species (14–100%). In general, these findings were actually consistent with other studies that reported moderate prevalence in wild aquatic turtles (44, 45, 70, 74). Variation in *Salmonella* prevalence reported in past studies could be attributed to differences in sample collection (39, 42) or culture methodology (23, 75–77). Depending on sample type, the use and type of *Salmonella* enrichment media results in significant differences in isolation efficacy (75–77) and secondary enrichment can significantly increase *Salmonella* isolation (23). Variations may also be related to species-specific susceptibility to *Salmonella* infection (39), habitat type (39), geographic location (20, 78, 79), and/or degree of anthropogenic influence (e.g., sewage or agricultural runoff) (80–82) on water bodies.

There were significant differences in *Salmonella* prevalence by turtle species. Painted turtles and sliders had significantly lower prevalence rates compared to snapping turtles, which may be attributable to undetermined ecological differences. Although all of the spiny softshell turtles were colonized by *Salmonella* (100%), these results should be interpreted with caution because the sample size of this species was very small (*n* = 4) and all four turtles came from the same pond (Lake Herrick). Interestingly, bottom-dwelling species (musk and snapping turtles) had a higher prevalence than basking species. Gaertner et al. (44) also found a higher prevalence of *Salmonella* in cloacal swabs of non-basking turtles [12/19 (63%), musk and snapping turtles)] compared with basking turtles [8/30 (27%), red-eared sliders and Texas river cooters (*Pseudemys texana*)]. Research is needed to determine whether this is due to exposure (e.g., *Salmonella* settling in pond detritus) or the effects of higher temperatures on turtles' immune system function, as behavioral basking is associated with increased immune system activity in ectotherms (83). A statistically-significantly higher prevalence of *Salmonella* infection occurred in juveniles than adults. This follows the general pattern in other animals of *Salmonella* infection in juveniles vs. adults, due to immature immune systems or gastrointestinal microflora (37). In addition, juvenile turtles may have a higher probability of *Salmonella* exposure through their more omnivorous diet, may spend more time hiding in detritus, or may be being more easily stressed than adults, which may increase susceptibility or shedding. As expected, there was a trend toward increasing *Salmonella* prevalence with higher ambient temperature, inferred from seasonal monthly data. Higher temperatures create a favorable environment for *Salmonella*, and previous studies have shown that *Salmonella* isolations from water bodies increased during summer months due to enhanced environmental persistence and replication of the bacteria, as well as increased storm events that flush more bacteria into river systems or stir up sediment (20).

Lastly, although the most plausible model found in model selection included only the effect of basking class, other models in the candidate set were found to be competing models, with delta AIC of <2. Competing models represent other plausible explanations for the dataset. These four competing models all included the effect of basking class, but each also included a landscape variable, meaning that these variables also likely explain some of the variation in *Salmonella* prevalence. For example, one competing model included the effect of basking class and percent of forest cover in the landscape, and a second included the effect of basking class and the canopy cover above the pond. A third and fourth competing model included the effect of basking class with the distance from streets and highways. In general, the prevalence of *Salmonella* decreased as the percent of forest and canopy cover and the distance from streets and highways increased and *Salmonella* prevalence increased as the percent of low-density residential areas increased. These results are consistent with the general theory that anthropogenic modifications to the landscape affect *Salmonella* contamination of water bodies (84, 85).

The nine serovars isolated from the majority of turtles included those commonly reported in reptiles (e.g., *S*. Java and *S*. Arizonae) (86), but they also included other types not historically reported commonly in turtles (e.g., *S*. Montevideo, *S*. Newport). The isolation of these atypical serovars suggests that turtles could be colonized with *S. enterica* serotypes that are concurrently present in the water body, possibly because of anthropogenic influences (20, 21, 78, 79). Zoomorphic variables (vicinity of poultry or cattle farms, animal manure application to pastureland, etc.) might also factor into environmental contamination of the turtles' habitat (78). While our sampling sites were in the mostly suburban area of Athens, GA, poultry farms occur along the Oconee River at the northern and western part of this watershed. Application of poultry manures to pastureland is also a common practice and runoff from these fields could find its way to this watershed (87). Many of these turtle isolates appear to be pathogenic for humans, given that 86% of these isolates matched PulseNet PFGE patterns of isolates from humans. In addition, a significantly higher proportion of turtle *Salmonella* isolates matched PFGE patterns of human salmonellosis cases, compared to the proportion of isolates from other animals and water bodies in the Oconee River watershed that matched human salmonellosis cases (84.8 vs. 50%, mostly comprised of mesomammals [e.g., raccoons (*Procyon lotor*) and Virginia opossums (*Didelphis virginianus*)] utilizing water bodies (21). While temporally there is considerable diversity in *Salmonella* serovar and strain types present in the Oconee River watershed, there are specific serovars and strain types that are repeatedly detected (21, 66). One serovar, *S*. Rubislaw, is of particular significance; this serovar is increasing in frequency in humans in Georgia and across the Southeastern United States (49, 88). Despite its strain type diversity, there was a significantly higher proportion of *S*. Rubislaw from turtles that matched human PulseNet PFGE patterns than Rubislaw isolated from other animals and water in the Oconee River watershed (21). While many of the *Salmonella* PFGE patterns in turtle isolates matched PulseNet patterns associated with outbreaks, temporal and spatial overlap between *Salmonella* isolation from turtles and humans was not as strong as similar studies (7, 10, 11, 21). It is likely turtles have acquired *Salmonella* from a human source (e.g., wastewater). A large number of aging septic systems and sewer lines may contribute to surface water contamination, including where turtles are found (89, 90).

The higher prevalence in turtles of PFGE patterns associated with human clinical disease may be because turtles inherently have more contact with water than mesomammals and birds (21), and sampling water requires collecting and filtering large amounts of water for testing. Turtles may accumulate *Salmonella* from their environment at higher levels that are easier to detect than in water alone. This accumulation could have important practical applications for better estimating bacterial contamination in the environment. For example, *Salmonella* bacteria in irrigation ponds have been identified as one likely source of contamination of produce (91); yet, the generally low detection rate of *Salmonella* in these aquatic environments (92) poses an epidemiological challenge for identifying the environmental sources of produce-associated outbreaks. These results suggest that sampling turtles in these possibly source environments might be more efficient than sampling the water bodies themselves. Lastly, whole genome sequencing has very recently replaced PFGE for PulseNet comparisons of isolates and is recommended for future studies.

In conclusion, there is significant overlap in the *S. enterica* serovars and strains that are associated with both wild turtles and human populations in Georgia. Whether the turtles are the source of human cases or just a sentinel of environmental contamination is currently unknown. Reptile-associated salmonellosis remains a public health concern (4, 7, 8, 28, 35, 93, 94). The current investigation by the CDC of an ongoing outbreak of salmonellosis linking human infections with contact with infected wild birds or contaminated feeders should serve as a reminder of the significance of understanding salmonellae dynamics in wildlife (95). There is high variability in the rates of *S. enterica* shedding among turtles but to better understand the epidemiology of *Salmonella* in turtles in the United States, a large-scale, nationwide study investigating the *Salmonella* prevalence of healthy, asymptomatic free-living turtles is needed. Of note, the data in this study were acquired in 2012–2013 so contemporary studies are needed to determine if the epidemiologic patterns observed are consistent. However, since this study was conducted, there have been no additional studies on *Salmonella* in turtles in Georgia or the Southeastern United States, so these data provide the most recent, but historic prospective on prevalence and epidemiology factors related to turtle infections which can guide future studies. Despite the age of these data, we suggest that turtles could be excellent indicators for levels of *Salmonella* contamination in those water bodies because turtles spend most of their lives in often poor-quality ponds, acquire *Salmonella* from those water bodies, and are relatively easy to attract and capture with baited traps.

## Data Availability Statement

The original contributions presented in the study are included in the article/Supplementary Material, further inquiries can be directed to the corresponding author/s.

## Ethics Statement

The animal study was reviewed and approved by University of Georgia's Institutional Animal Care and Use Committee (AUP# A2010 10-186).

## Author Contributions

SH, MY, and JM contributed to study design, collected data, and wrote manuscript. VP conducted statistical analyses and contributed to the manuscript. JM, KH, PG-S, and AP analyzed and interpreted data and contributed to the manuscript. JH, KJ, and TK collected data. SS, PG-S, and KH analyzed, interpreted data, and contributed to manuscript revisions. KJ and TK analyzed and interpreted data. JM, EL, MM, and SC contributed to manuscript revisions. EL analyzed data, contributed funds, and contributed to the manuscript. All authors contributed to the article and approved the submitted version.

## Conflict of Interest

The authors declare that the research was conducted in the absence of any commercial or financial relationships that could be construed as a potential conflict of interest.
